# Deletion of Metabotropic Glutamate Receptors 2 and 3 (mGlu2 & mGlu3) in Mice Disrupts Sleep and Wheel-Running Activity, and Increases the Sensitivity of the Circadian System to Light

**DOI:** 10.1371/journal.pone.0125523

**Published:** 2015-05-07

**Authors:** David Pritchett, Aarti Jagannath, Laurence A. Brown, Shu K. E. Tam, Sibah Hasan, Silvia Gatti, Paul J. Harrison, David M. Bannerman, Russell G. Foster, Stuart N. Peirson

**Affiliations:** 1 Nuffield Department of Clinical Neurosciences (Nuffield Laboratory of Ophthalmology), University of Oxford, John Radcliffe Hospital, Oxford, OX3 9DU, United Kingdom; 2 F.Hoffman-La Roche, Neuroscience, Ophthalmology & Rare Diseases (NORD), Pharma Research & Early Development (pRED) Innovation Centre, Basel, Switzerland; 3 Department of Psychiatry, University of Oxford, Warneford Hospital, Oxford, OX3 7JX, United Kingdom; 4 Department of Experimental Psychology, University of Oxford, South Parks Road, Oxford, OX1 3UD, United Kingdom; Kent State University, UNITED STATES

## Abstract

Sleep and/or circadian rhythm disruption (SCRD) is seen in up to 80% of schizophrenia patients. The co-morbidity of schizophrenia and SCRD may in part stem from dysfunction in common brain mechanisms, which include the glutamate system, and in particular, the group II metabotropic glutamate receptors mGlu2 and mGlu3 (encoded by the genes *Grm2* and *Grm3*). These receptors are relevant to the pathophysiology and potential treatment of schizophrenia, and have also been implicated in sleep and circadian function. In the present study, we characterised the sleep and circadian rhythms of *Grm2/3* double knockout (*Grm2/3* ^-/-^) mice, to provide further evidence for the involvement of group II metabotropic glutamate receptors in the regulation of sleep and circadian rhythms. We report several novel findings. Firstly, *Grm2/3* ^-/-^ mice demonstrated a decrease in immobility-determined sleep time and an increase in immobility-determined sleep fragmentation. Secondly, *Grm2/3* ^-/-^ mice showed heightened sensitivity to the circadian effects of light, manifested as increased period lengthening in constant light, and greater phase delays in response to nocturnal light pulses. Greater light-induced phase delays were also exhibited by wildtype C57Bl/6J mice following administration of the mGlu2/3 negative allosteric modulator RO4432717. These results confirm the involvement of group II metabotropic glutamate receptors in photic entrainment and sleep regulation pathways. Finally, the diurnal wheel-running rhythms of *Grm2/3* ^-/-^ mice were perturbed under a standard light/dark cycle, but their diurnal rest-activity rhythms were unaltered in cages lacking running wheels, as determined with passive infrared motion detectors. Hence, when assessing the diurnal rest-activity rhythms of mice, the choice of assay can have a major bearing on the results obtained.

## Introduction

Schizophrenia is a debilitating psychiatric disorder that affects 0.3–0.7% of the world’s population [1], while sleep and/or circadian rhythm disruption (SCRD) is observed in 30–80% of schizophrenia patients [2]. Sleep disturbances in schizophrenia include increases in sleep latency and sleep fragmentation, and reductions in total sleep time, sleep efficiency, rapid eye movement (REM) sleep latency, REM sleep density and slow-wave sleep duration [2–4]. Typical circadian abnormalities in the disorder include the abnormal phasing, instability and fragmentation of rest-activity rhythms [5–9]. Some of these abnormalities may be secondary to the disorder and its drug treatment, but the co-morbidity of schizophrenia and SCRD may also stem from dysfunction in common brain mechanisms (e.g. specific neurotransmitter systems) [10, 11].

The glutamate system is one such candidate mechanism. There is strong evidence of glutamatergic dysfunction in schizophrenia [12, 13], while glutamate is the principal neurotransmitter within the retinohypothalamic tract (RHT), which conveys photic entrainment cues from the retina to the circadian timing system in the suprachiasmatic nucleus (SCN) [14–16]. Glutamatergic signalling is also important for bilateral communication between the left and right SCN [17], while the SCN stimulates the paraventricular and ventrolateral preoptic nuclei via glutamatergic efferents [18–22]. Glutamate release in the prefrontal cortex displays rhythmic fluctuations during the sleep-wake cycle; it increases during wakefulness and REM sleep episodes, but decreases during non-REM sleep [23–25].

At the receptor level, group II metabotropic glutamate receptors may be particularly relevant to the pathophysiology of schizophrenia. mGlu2 and mGlu3, encoded by the genes *Grm2* and *Grm3*, are presynaptic inhibitory autoreceptors that are widely expressed in both rodent and human brain [26–31]. *Grm2* expression is predominantly neuronal, whereas *Grm3* expression is primarily glial [28]. In a recent meta-analysis of genome-wide association studies, a strong association was reported between the *Grm3* locus and schizophrenia [32], extending prior evidence that *Grm3* is involved in the genetic predisposition to psychosis [33–36]. Polymorphic variation in *Grm3* has also been associated with cognitive performance, prefrontal glutamatergic neurotransmission and hippocampal pathophysiology [33, 37]. In addition, there is clinical and preclinical evidence that mGlu2 & 3 may be therapeutic targets for the disorder, although no drug in this category has yet been licensed for use [38].

There is also increasing evidence that implicates group II metabotropic glutamate receptors in sleep and circadian function. Firstly, *Grm2* and *Grm3* are expressed in the SCN [14, 39]. mGlu2 & 3 modulate NMDA-evoked calcium responses in SCN neurons, hinting at a possible role in photic entrainment [40]. Consistent with this, mGlu2/3-modulating drugs influence the magnitude of light-induced phase delays in the rest-activity rhythms of wildtype hamsters [41, 42]. Secondly, the administration of mGlu2/3-modulating drugs profoundly alters sleep structure in wildtype rats; both agonists and antagonists trigger a selective suppression of REM sleep [43–50]. Thirdly, an mGlu2/3 antagonist and an mGlu2 negative allosteric modulator increase sleep fragmentation in wildtype rats [44]. Lastly, mGlu2 and/or mGlu3 may regulate the inhibitory output of the thalamic reticular nucleus, which forms part of the ascending arousal system [51, 52].

In the present study, we characterised the sleep and circadian rhythms of *Grm2/3* double knockout (*Grm2/3* ^-/-^) mice. Although expression of the dimeric form of mGlu3 is reduced in the brains of schizophrenia patients [53], mGlu2 and mGlu3 are not completely absent, so the *Grm2/3* ^-/-^ mouse cannot be considered a ‘disease model’. Instead, our primary aim was to obtain additional evidence for the involvement of group II metabotropic glutamate receptors in the regulation of sleep and circadian rhythms. Based on the evidence described above, we predicted that *Grm2/3* ^-/-^ mice would demonstrate a reduction in total sleep duration, increased sleep fragmentation, and heightened sensitivity to the circadian effects of light (e.g. light-induced phase shifts of greater magnitude).

## Materials and Methods

### Animals

We elected to study *Grm2/3* double knockout mice because the behavioural phenotypes of *Grm2* and *Grm3* single knockout mice are mild in comparison [54, 55]. This may be due to the fact that the deletion of *Grm3* results in a compensatory upregulation in the expression of *Grm2*, and vice versa [56]. The use of the double knockout also enables us to make direct comparisons with the pharmacological literature, wherein most studies used drugs that act on mGlu2 and mGlu3 indiscriminately [43–50].

*Grm2/3* double knockout (*Grm2/3* ^-/-^) mice were obtained from GlaxoSmithKline (Harlow, UK). These mice were generated as described previously [55]. Briefly, *Grm2* knockout mice (*Grm2* ^-/-^) [57] were crossed with *Grm3* knockout mice (*Grm3* ^-/-^) [58] to generate double heterozygous (*Grm2* ^+/-^ *Grm3* ^+/-^) offspring. (Both single knockout models were on a C57Bl/6 background). Double heterozygous mice were then crossed to generate 1:16 double knockout mice (*Grm2/3* ^-/-^), 1:16 wildtype mice (*Grm2/3* ^+/+^), and 14:16 mice that were heterozygous for *Grm2* and/or *Grm3*. Separate lines of *Grm2/3* ^-/-^ and *Grm2/3* ^+/+^ mice were subsequently established to avoid the prohibitive wastage of animals.

Age-matched males were used in all experiments. Mice were at least 2 months old at the onset of behavioural screening and were no older than 9 months upon completion of testing. Only males were tested due to the potentially confounding influence of the oestrus cycle on wheel-running activity [59]. 2 transgenic cohorts were used; cohort 1 consisted of 11 *Grm2/3* ^+/+^ mice and 12 *Grm2/3* ^-/-^ mice, while cohort 2 was comprised of 12 mice of each genotype. For the experiment involving the administration of the drug RO4432717 (F.Hoffmann-LaRoche, Basel, Switzerland), a separate cohort of 22 male wildtype C57Bl/6J mice was used (Harlan, UK). Mice were singly-housed and had access to food and water *ad libitum*. All behavioural procedures were performed in accordance with the United Kingdom Animals (Scientific Procedures) Act of 1986 and the University of Oxford Policy on the Use of Animals in Scientific Research. All experiments were approved by the University of Oxford Animal Welfare and Ethical Review Board, and were conducted under the PPL 30/2812 by PILs 30/9339 and 30/8553.

### Order of tests

Cohorts 1 and 2 completed an extensive wheel-running screen consisting of several standard protocols (full details below). Video-tracking, passive-infrared (PIR) motion detection and object interaction testing were only performed on cohort 2. The order of these tests was counterbalanced across genotype groups. Half of cohort 2 underwent video-tracking followed by PIR motion detection, then wheel-running, and finally object interaction testing. The other half of cohort 2 were subjected to PIR motion detection followed by video-tracking, then object interaction testing, and finally wheel-running.

### Diurnal rest-activity rhythm and sleep screening

#### Video-tracking

Video-tracking analyses were based on an established protocol [60]. Mice were individually housed in large cages (44 cm long × 26 cm wide × 12 cm high), positioned within light-tight chambers (LTCs), illuminated by an overhead cool white LED light-source. LTCs were maintained at constant temperature (21°C) and humidity (50%). Each LTC contained 6 cages, with 3 mice of each genotype housed in alternating positions. A standard 12:12 h light/dark (12:12 LD) cycle was employed and illuminance was 100 lux at the base of each cage. Three 24 h videos (i.e. 3 separate 12:12 LD cycles) were recorded at weekly intervals, using a near-infrared CCTV camera (Maplin Electronics, Rotherham, UK). All videos were recorded at 3 frames per second, in the AVI file format. An acrylic block was placed under the food hopper to keep the mouse in the recording field at all times.

Video files were stored on a digital hard drive recorder (Samsung, Suwon, South Korea) prior to analysis with ANY-maze 4.5 (Stoelting, Wood Dale, Illinois). Multiple immobility-determined sleep parameters were extracted from the video footage: total sleep time, light and dark phase sleep time, light and dark phase sleep bouts, and light and dark phase sleep bout duration. Sleep was defined as a period of immobility of at least 40 s, a previously established proxy measure of sleep [60]. This measure has an extremely high concordance (>95%) with EEG-based sleep determination [60]. Immobility sensitivity was set at 95% to prevent the detection of movements caused by breathing during sleep.

In addition to the sleep parameters outlined above, four activity parameters were extracted from the video footage using ANY-maze 4.5: total activity, light and dark phase activity (distance travelled in metres), and relative light phase activity (the percentage of total activity occurring during the light phase). Note that one *Grm2/3* ^+/+^ mouse was excluded from all dark phase sleep and activity analyses as it escaped from its cage during dark phase recording.

3–5 days after the third 24 h recording, mice were exposed to a 1 h 100 lux light pulse from zeitgeber time (ZT) 16 to 17. (ZT0 refers to the onset of the light phase, while ZT12 denotes the onset of the dark phase). Negative masking—percent activity suppression induced by the light pulse—was computed to provide an indication of photosensitivity [61]. This was calculated by comparing activity levels during the light pulse with average activity levels during the same time window (ZT16-17) across the previous 3 days.

#### Passive-infrared (PIR) motion detection

Due to the prohibitive size of the video files, it was impractical to analyse more than 72 h of video data. PIR motion detection provides a less data-heavy alternative, enabling the estimation of sleep over a longer period; in this case, 14 consecutive days of 12:12 LD at 100 lux. It also enables the computation of specific parameters that cannot be extracted from isolated 24 h video recordings, such as period length (see below). Full details of this novel technique, developed in our laboratory, should be published in the near future [Brown *et al*., *in review*]. Housing conditions for the PIR protocol were identical to that of the video-tracking protocol outlined above. Again, an acrylic block was placed under the food hopper to keep the mouse in the motion detection field at all times. Raw data from the PIR motion sensors took the form of % time active per 10 s epoch, with sensors activated both by gross locomotion and small movements such as turning of the head. Three immobility-determined sleep parameters were extracted from the raw PIR data using Microsoft Excel 2013 (Microsoft, Redmond, USA): total sleep time, light phase sleep time and dark phase sleep time. Note that methods do not yet exist to automatically compute parameters pertaining to the frequency and duration of sleep bouts from PIR data. As with the previously described video-based method of sleep determination [60], sleep was defined as a period of immobility of at least 40 s. We have recently confirmed that this measure has an extremely high concordance (>95%) with both video-based sleep determination and EEG-based sleep determination [Brown *et al*., *in review*].

In addition to the sleep parameters outlined above, four activity parameters were extracted from the raw PIR data using the ClockLab toolbox (Actimetrics, Illinois, USA) for MatLab (MathWorks, Massachusetts, USA): total activity, light and dark phase activity (expressed in arbitrary units), and relative light phase activity (as above).

Standard diurnal rest-activity rhythm parameters were also computed from the raw PIR data using ClockLab; these comprised two measures of diurnal rest-activity rhythm fragmentation (daily activity bouts and daily activity bout duration), two measures of diurnal rest-activity rhythm consistency (chi-square periodogram amplitude and onset tau error), and period length. Activity bouts were defined using pre-established criteria for ClockLab (maximum gap: 18 min; threshold: 5%). A sixth diurnal rest-activity rhythm parameter—interdaily stability—was computed using the software package Actiwatch Activity & Sleep Analysis 7 (Cambridge Neurotechnology, Cambridge, UK). Interdaily stability is a non-parametric measure that quantifies the similarity of activity patterns across multiple days [62].

### Diurnal rest-activity rhythm and circadian screening

#### Wheel-running

This screen was based on an established protocol [63–65]. Housing conditions were identical to that of the video-tracking and PIR protocols described above, except that cages were fitted with running wheels (18 cm diameter). As previously, illuminance was 100 lux at the base of each cage. Mice in both cohorts were exposed to 14 days of 12:12 LD and 11 days of constant dark (DD). In addition, cohort 1 was subjected to 13 days of constant light (LL), and a 6 h phase advance during 12:12 LD.

During 12:12 LD, four activity parameters were extracted from the raw wheel-running data using ClockLab: total activity, light and dark phase activity (wheel rotations), and relative light phase activity (as above). The six diurnal rest-activity rhythm parameters computed from the raw PIR data (see above) were also computed from the raw wheel-running data, using ClockLab and Actiwatch Activity & Sleep Analysis 7. Again, activity bouts were defined using pre-established criteria (maximum gap: 18 min; threshold: 5 counts/min). Note that two *Grm2/3* ^-/-^ mice were omitted from these activity bout analyses as their activity levels were too low to derive bout data. ClockLab was also used to quantify period length under free-running conditions (i.e. DD and LL), while re-entrainment following the 6 h phase advance was scored manually from individual actograms as the number of days taken to re-entrain.

Both cohorts were subjected to type I and type II phase-shifting light pulses [66] (using previously described protocols [64, 67]) to induce a phase delay in their wheel-running rhythms, providing an indication of circadian photosensitivity. For the type I pulse, mice were exposed to 100 lux light for 15 minutes at circadian time (CT) 16 during DD. The magnitude of the phase delay was quantified by fitting one regression line through 10 consecutive activity onsets preceding the light pulse, and another through 6 consecutive activity onsets following the pulse. The first 2 activity onsets following the light pulse were disregarded because of possible transition effects [64]. For the type II pulse, mice were exposed to 100 lux light for one hour at ZT16 during a standard 12:12 LD cycle. Following the pulse, mice were released into DD. The magnitude of the phase delay was quantified by fitting one regression line through 6 consecutive activity onsets preceding the light pulse, and another through 6 consecutive activity onsets following the pulse. This time, only one activity onset following the light pulse was disregarded [64]. For both light pulses, the magnitude of the phase delay was calculated as the time difference between the two regression lines on the first day after the light pulse. Negative masking was also computed for the type II pulse, using the same method employed in the video-tracking screen.

### Administration of an mGlu2/3 negative allosteric modulator to wildtype mice prior to a type I phase-shifting light pulse

To further investigate the hypothesis that group II metabotropic glutamate receptors modulate photic entrainment, the mGlu2/3 negative allosteric modulator RO4432717 was administered to wildtype mice in a standard phase-shifting paradigm. The pharmacology of RO4432717 (previously known as compound 7i) is described elsewhere [68]. 22 male wildtype C57Bl/6J mice (aged 80 days) were individually housed in large cages (44 cm long × 26 cm wide × 12 cm high), fitted with running wheels (18 cm diameter), under a 12:12 LD cycle at 400 lux. All mice were kept in DD for 1 day prior to the administration of a 30 min type I phase-shifting light pulse (400 lux) at CT16. 3 mice underwent no pre-treatment, while 6 mice received an intraperitoneal injection of saline (0.3 ml) at CT12 on the day of the light pulse. The remaining 13 mice received an intraperitoneal injection of RO4432717 at CT12. Three dosages were used: 3 mg (4 mice), 10 mg (5 mice) and 30 mg (4 mice). Injection volumes were kept constant at 0.3 ml. The magnitude of the phase delay was calculated using the method described above.

### Home-cage object interaction test

To evaluate whether the altered wheel-running behaviour of *Grm2/3* ^-/-^ mice was a consequence of increased object neophobia (toward their running wheels), a simple object interaction task was conducted within the home-cage. The results of this test are presented in S1 Fig.

## Statistical Analyses

All statistical analyses were performed with SPSS 22.0 (IBM, Armonk, New York). Unless otherwise stated, all reported statistics are the result of analyses of variance, with genotype included as the principal independent variable. Differences were considered to be statistically significant at p-values < 0.05. Greenhouse-Geisser corrections were applied where appropriate, but uncorrected degrees of freedom are reported in order to preserve the transparency of the statistical design. For analyses involving both experimental cohorts, further analyses of variance were conducted with cohort included as an extra independent variable. However, no additional effects or interactions were observed. In all figures, * indicates a p-value ≤ 0.05, ** indicates a p-value ≤ 0.01, and *** indicates a p-value ≤ 0.001. Error bars depict the standard error of the mean. *M* = mean.

## Results

### *Grm2/3* ^-/-^ mice display reduced sleep time and increased sleep fragmentation

#### Video-tracking sleep analyses

**Fig 1:**
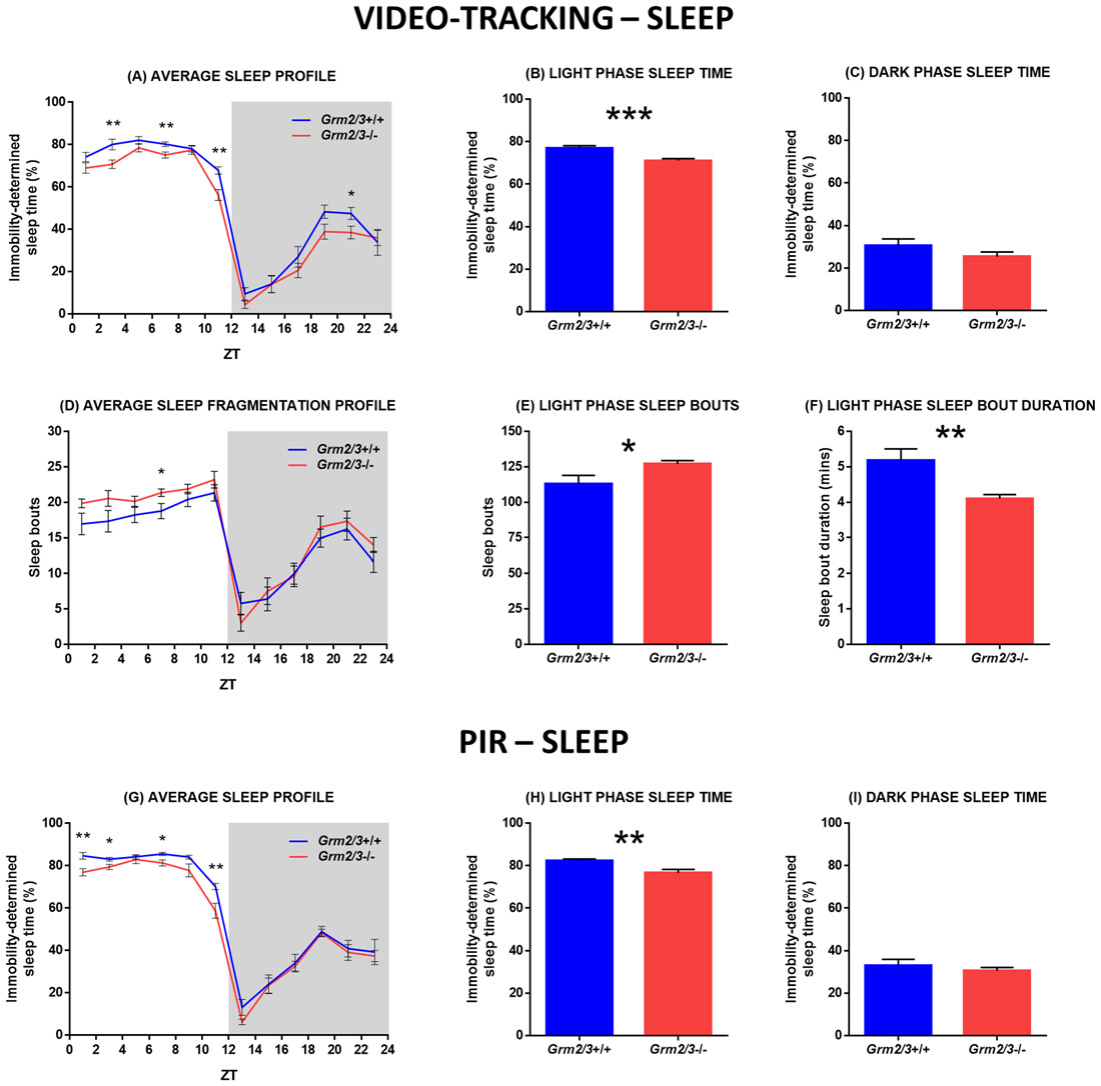
Sleep time is reduced and sleep fragmentation is increased in *Grm2/3* ^-/-^ mice. Fig 1A-F depict immobility-determined sleep parameters derived from 3 separate days of video-tracking data. Fig 1G-I depict immobility-determined sleep parameters derived from 14 consecutive days of passive-infrared (PIR) data. (A & G) Average sleep profiles, (B & H) light phase sleep time, and (C & I) dark phase sleep time for *Grm2/3* ^**+/+**^ and *Grm2/3* ^**-/-**^ mice during a 12:12 h light/dark (12:12 LD) cycle at 100 lux. (D) Average temporal distribution of sleep bouts, (E) number of light phase sleep bouts, and (F) light phase sleep bout duration in *Grm2/3* ^**+/+**^ and *Grm2/3* ^**-/-**^ mice during 12:12 LD at 100 lux. Sleep was defined as a period of immobility of at least 40 s. Data in Fig 1A, D & G are presented in 2 h time bins. ZT = zeitgeber time. Note that methods do not yet exist to automatically compute parameters pertaining to the frequency and duration of sleep bouts from PIR data.

Relative to *Grm2/3* ^+/+^ mice, total sleep time was markedly reduced in *Grm2/3* ^-/-^ mice (*F*_1,21_ = 6.456, P = 0.019; Fig 1A). This reduction in sleep time was driven by decreased light phase sleep (*F*_1,22_ = 18.107, P = <0.001; Fig 1B); on average, *Grm2/3* ^+/+^ mice spent 77.0% of the light phase asleep, while *Grm2/3* ^-/-^ mice slept for only 71.1% of the light phase. By contrast, dark phase sleep time did not vary according to genotype (*F*_1,21_ = 1.868, P = 0.186; Fig 1C).

Sleep during the light phase was not only reduced but also more fragmented in *Grm2/3* ^-/-^ mice (see Fig 1D); *Grm2/3* ^-/-^ mice demonstrated a greater number of light phase sleep bouts (*F*_1,22_ = 4.664, P = 0.042; Fig 1E) of a shorter average duration (*F*_1,22_ = 9.315, P = 0.006; Fig 1F). Dark phase sleep bouts were also shorter in *Grm2/3* ^-/-^ than *Grm2/3* ^+/+^ mice (*F*_1,21_ = 5.735, P = 0.026), although the total number of dark phase sleep bouts was unaffected by genotype (*F*_1,21_ = 0.050, P = 0.825). There were no effects or interactions involving recording day for any of these analyses, reflecting the fact that the sleep profiles of *Grm2/3* ^+/+^ and *Grm2/3* ^-/-^ mice were relatively consistent across the three recording sessions.

#### PIR sleep analyses

Over 14 days of continuous recording under 12:12 LD, light phase sleep time was reduced in *Grm2/3* ^-/-^ mice relative to *Grm2/3* ^+/+^ mice (*F*_1,22_ = 9.037, P = 0.007; Fig 1H). On average, *Grm2/3* ^+/+^ mice spent 82.3% of the light phase asleep, while *Grm2/3* ^-/-^ mice slept for only 76.6% of the light phase. There was also a trend towards a reduction in total sleep time (*F*_1,22_ = 3.510, P = 0.074; Fig 1G), but genotype had no effect on dark phase sleep time (*F*_1,22_ = 0.459, P = 0.505; Fig 1I).

### *Grm2/3* ^-/-^ mice are more sensitive to the circadian effects of light

#### Wheel-running analyses: Period length in constant dark (DD) and constant light (LL)

**Fig 2:**
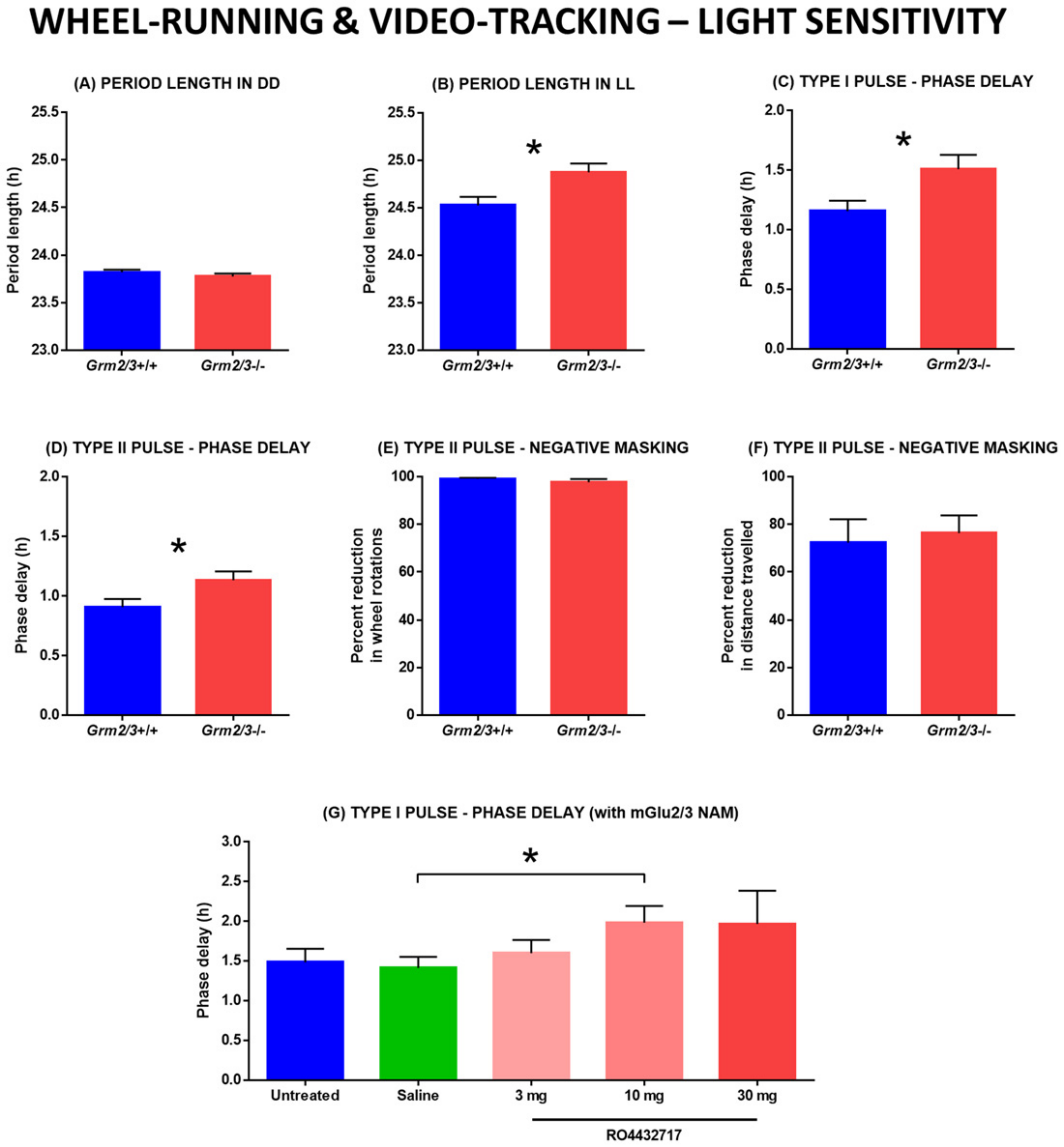
Light sensitivity is increased by the genetic ablation or pharmacological inhibition of mGlu2 & 3. All figures depict rest-activity or circadian parameters derived from wheel-running data, with the exception of Fig 2F, which is based on video-tracking data. (A) Free-running period length in constant dark (DD) does not differ between *Grm2/3* ^**+/+**^ and *Grm2/3* ^**-/-**^ mice. (B) Free-running period length in constant light (LL) is greater in *Grm2/3* ^**-/-**^ than *Grm2/3* ^**+/+**^ mice. (C & D) Phase delays induced by type I (Fig 2C) and type II (Fig 2D) phase-shifting light pulses are larger in *Grm2/3* ^**-/-**^ than *Grm2/3* ^**+/+**^ mice. (E & F) *Grm2/3* ^**+/+**^ and *Grm2/3* ^**-/-**^ mice show similar levels of negative masking during a type II light pulse, as assayed by wheel-running (Fig 2E) and video-tracking (Fig 2F). (G) In wildtype C57Bl/6J mice, phase delays induced by a type I light pulse are enhanced following the administration of the mGlu2/3 negative allosteric modulator (NAM) RO4432717.

Over 11 days of continuous recording under DD, genotype had no effect on free-running period length (*F*_1,45_ = 1.159, P = 0.287; Fig 2A); average period length was 23.82 h in *Grm2/3* ^+/+^ mice and 23.78 h in *Grm2/3* ^-/-^ mice. By contrast, period length was considerably longer in *Grm2/3* ^-/-^ than *Grm2/3* ^+/+^ mice over 13 days of continuous recording under LL (*F*_1,21_ = 7.242, P = 0.014; Fig 2B); average period length was 24.87 h in *Grm2/3* ^-/-^ mice, but only 24.53 h in *Grm2/3* ^+/+^ mice.

#### Wheel-running analyses: Phase delays induced by nocturnal light pulses

A type I phase-shifting light pulse at CT16 produced larger phase delays in the rest-activity rhythms of *Grm2/3* ^-/-^ than *Grm2/3* ^+/+^ mice (*F*_1,44_ = 5.498, P = 0.024; Fig 2C); the average phase delay was 1.51 h in *Grm2/3* ^-/-^ mice, but only 1.16 h in *Grm2/3* ^+/+^ mice. A type II phase-shifting light pulse at ZT16 also yielded larger phase delays in the rest-activity rhythms of *Grm2/3* ^-/-^ than *Grm2/3* ^+/+^ mice (*F*_1,45_ = 4.736, P = 0.035; Fig 2D); the average phase delay was 1.13 h in *Grm2/3* ^-/-^ mice, but only 0.91 h in *Grm2/3* ^+/+^ mice.

#### Wheel-running and video-tracking analyses: Negative masking during nocturnal light pulses

Negative masking was unaffected by genotype, regardless of the method used to assay locomotor activity. Genotype had no effect on the degree of activity suppression induced by a type II light pulse in either the wheel-running screen (*F*_1,45_ = 0.994, P = 0.324; Fig 2E) or the video-tracking screen (*F*_1,22_ = 0.111, P = 0.743; Fig 2F).

### Wildtype mice are more sensitive to the circadian effects of light following the administration of an mGlu2/3 negative allosteric modulator

To discount the possibility that the increased light sensitivity of *Grm2/3* ^-/-^ mice is a consequence of altered neurodevelopment, we evaluated the impact of the mGlu2/3 negative allosteric modulator RO4432717 on the phase-shifting responses of wildtype C57Bl/6J mice. There was no difference in the magnitude of phase delays (induced by a type I light pulse at CT16) between untreated wildtype mice and wildtype mice injected with saline at CT12 (*F*_1,7_ = 0.096, P = 0.766; Fig 2G). Relative to saline-treated mice, phase delays were greater in mice injected with RO4432717 at CT12. This difference was statistically significant at the 10 mg dosage (*F*_1,9_ = 5.406, P = 0.045), but not at the 3 mg dosage (*F*_1,8_ = 0.724, P = 0.420) or 30 mg dosage (*F*_1,8_ = 2.194, P = 0.177). The 30 mg dose yielded large, but more variable effects.

### The diurnal rest-activity rhythms of *Grm2/3* ^-/-^ mice are dependent on the assay used to measure locomotor behaviour

#### Wheel-running activity analyses

**Fig 3:**
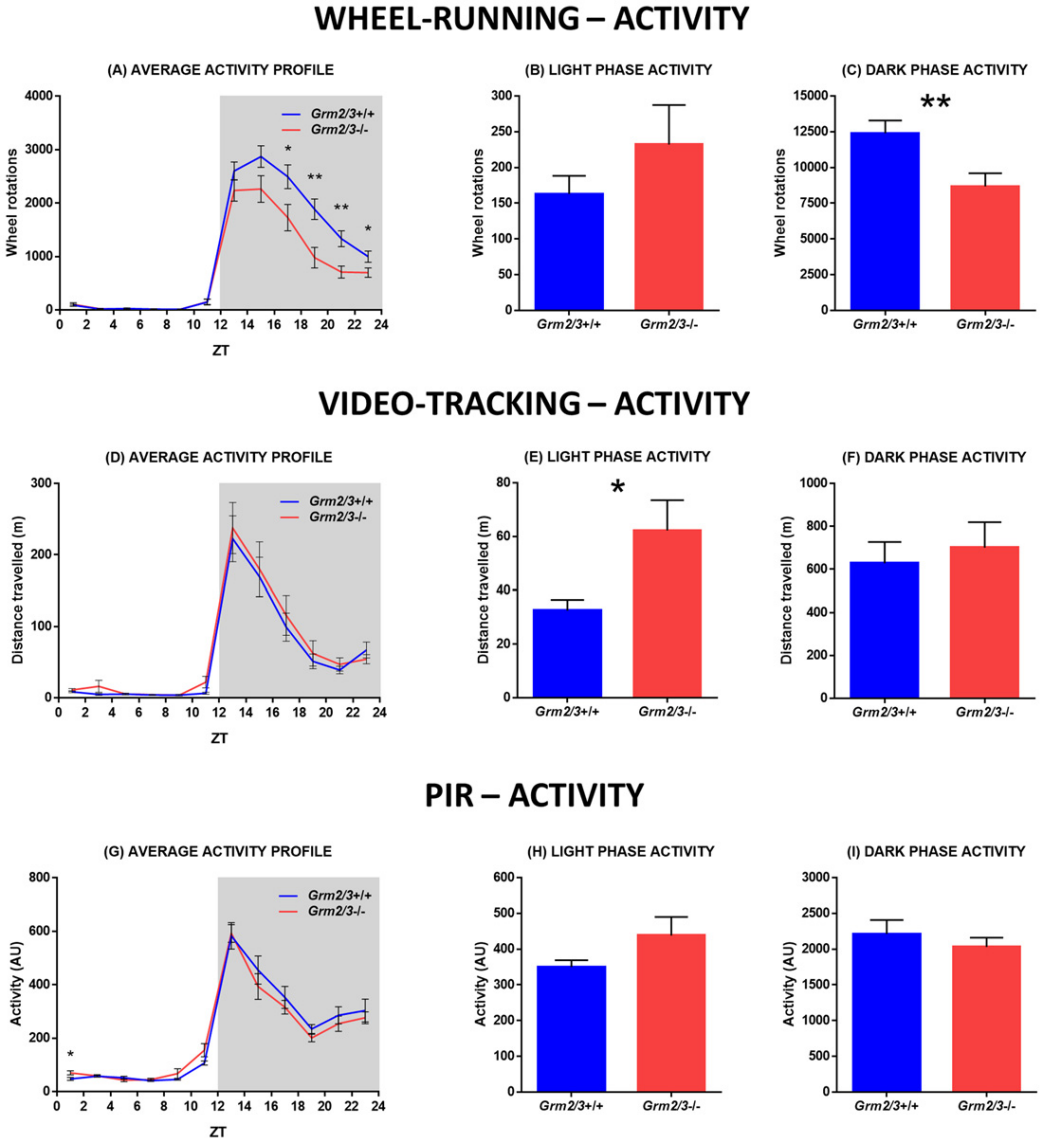
Dark phase wheel-running activity is reduced in Grm2/3^-/-^ mice, but general home-cage activity is not. Fig 3A-C depict activity parameters derived from 14 consecutive days of wheel-running data. Fig 3D-F depict activity parameters derived from 3 separate days of video-tracking data. Fig 3G-I depict activity parameters derived from 14 consecutive days of passive-infrared (PIR) data. (A, D & G) Average activity profiles, (B, E & H) light phase activity, and (C, F & I) dark phase activity for *Grm2/3* ^**+/+**^ and *Grm2/3* ^**-/-**^ mice during a 12:12 h light/dark (12:12 LD) cycle at 100 lux. Data in Fig 3A, D & G are presented in 2 h time bins. ZT = zeitgeber time. AU = arbitrary units.

**Fig 4:**
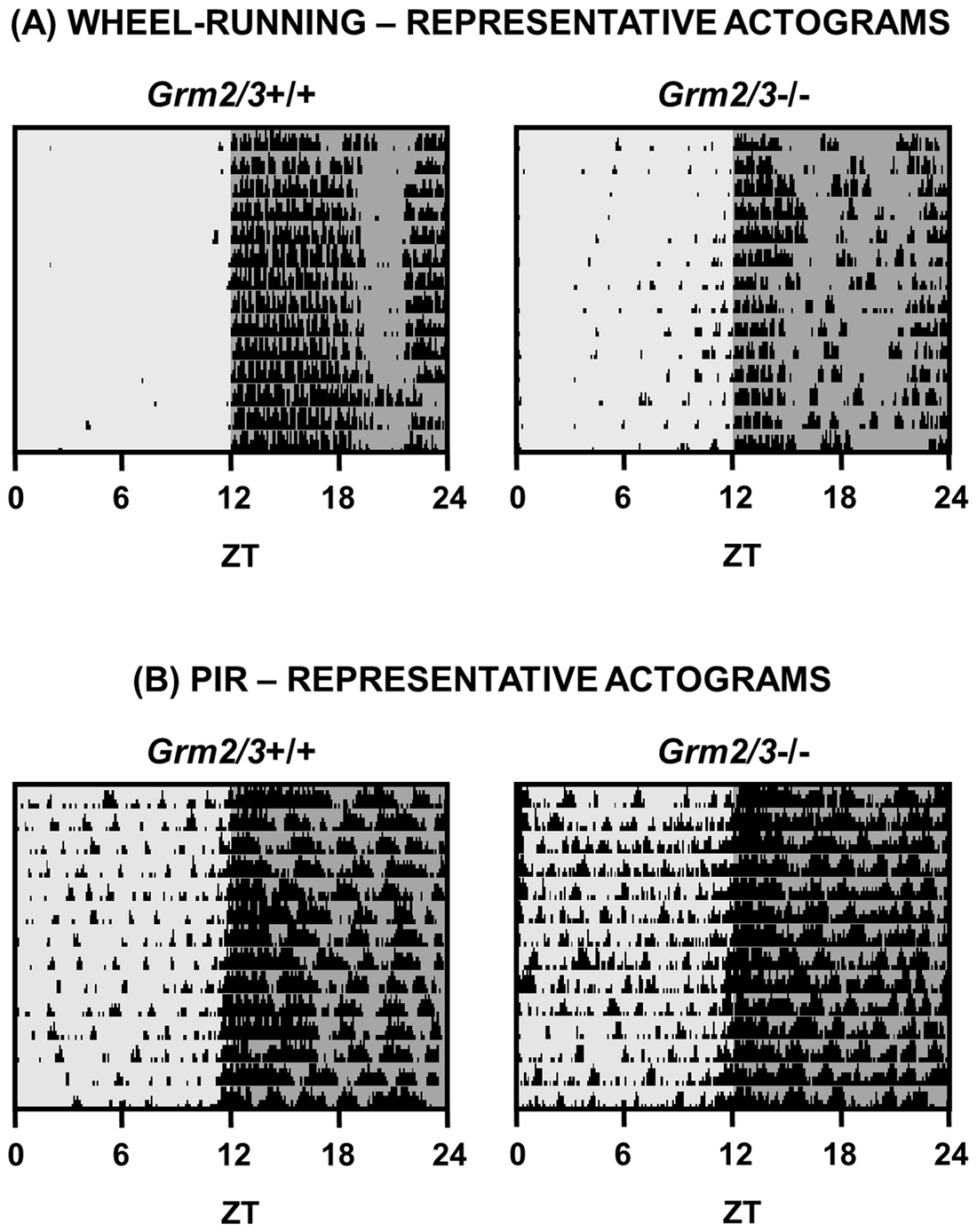
Representative actograms of *Grm2/3* ^+/+^ and *Grm2/3* ^-/-^ mice during a 12:12 h light/dark (12:12 LD) cycle. Each row depicts a single 24 h period. The light and dark grey shading corresponds to periods of (100 lux) light and dark, respectively. (A) Representative wheel-running actograms. The black bars denote periods of wheel-running activity, binned in 6 min epochs. The height of the bars corresponds to the number of wheel rotations within each epoch. (B) Representative passive-infrared (PIR) actograms. The black bars denote periods of home-cage activity, binned in 6 min epochs. The height of the bars corresponds to % time active within each epoch. ZT = zeitgeber time.

Over 14 days of continuous recording under 12:12 LD, total wheel-running activity was markedly reduced in *Grm2/3* ^-/-^ mice relative to *Grm2/3* ^+/+^ mice (*F*_1,45_ = 8.165, P = 0.006; Fig 3A). This reflects the fact that *Grm2/3* ^-/-^ mice were significantly less active than *Grm2/3* ^+/+^ mice during the dark phase (*F*_1,45_ = 8.503, P = 0.006; Fig 3C). By contrast, genotype had no effect on activity during the light phase (*F*_1,45_ = 1.257, P = 0.268; Fig 3B); indeed, numerically speaking, *Grm2/3* ^-/-^ mice were *more* active than *Grm2/3* ^+/+^ mice during the light phase. Consequently, the percentage of total daily activity which occurred during the light phase was greater in *Grm2/3* ^-/-^ mice than *Grm2/3* ^+/+^ mice (*F*_1,45_ = 7.585, P = 0.008). (Note that mean values for all wheel-running analyses are included in Table 1, and representative actograms of 12:12 LD are depicted in Fig 4A).

**Table 1:** Descriptive statistics for selected rest-activity parameters derived from 14 consecutive days of wheel-running data.

Parameter	*Grm2/3* ^+/+^ (mean ± SEM)	*Grm2/3* ^-/-^ (mean ± SEM)	p-value
Daily activity (wheel rotations)	12571 ± 898 (*n* = 23)	8914 ± 910 (*n* = 24)	0.006
Dark phase activity (wheel rotations)	12408 ± 890 (*n* = 23)	8682 ± 915 (*n* = 24)	0.006
Light phase activity (wheel rotations)	163 ± 25 (*n* = 23)	232 ± 55 (*n* = 24)	0.268
Relative light phase activity (%)	1.4 ± 0.2 (*n* = 23)	3.6 ± 0.8 (*n* = 24)	0.008
Daily activity bouts	3.5 ± 0.3 (*n* = 23)	4.4 ± 0.3 (*n* = 22)	0.029
Activity bout duration (min)	175 ± 15 (*n* = 23)	114 ± 14 (*n* = 22)	0.004
Interdaily stability (AU)	1.07 ± 0.05 (*n* = 23)	0.81 ± 0.06 (*n* = 24)	0.003
Chi-square periodogram amplitude (AU)	1841 ± 70 (*n* = 23)	1553 ± 111 (*n* = 24)	0.035
Onset tau error (h)	0.16 ± 0.05 (*n* = 23)	0.48 ± 0.16 (*n* = 24)	0.071
Period length (h)	23.99 ± 0.01 (*n* = 23)	23.99 ± 0.02 (*n* = 24)	0.922

Mice were housed under a 12:12 h light/dark (12:12 LD) cycle at 100 lux. Units of measurement and sample sizes are indicated in brackets. AU = arbitrary units. SEM = standard error of the mean.

**Fig 5:**
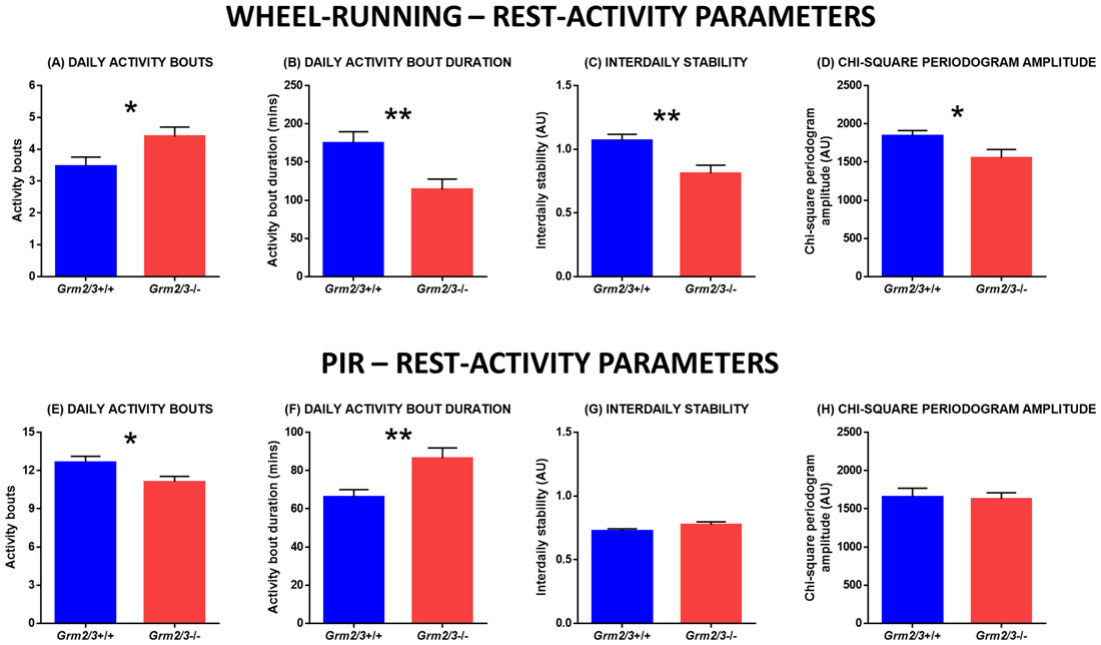
The diurnal rest-activity rhythms of *Grm2/3* ^-/-^ mice are dependent on the assay used to measure activity. Fig 4A–4D depict rest-activity parameters derived from 14 consecutive days of wheel-running data. Fig 4E–4H depict rest-activity parameters derived from 14 consecutive days of passive-infrared (PIR) data. (A & E) Daily activity bouts, (B & F) daily activity bout duration, (C & G) interdaily stability, and (D & H) chi-square periodogram amplitude in *Grm2/3* ^**+/+**^ and *Grm2/3* ^**-/-**^ mice during a 12:12 h light/dark (12:12 LD) cycle at 100 lux. Activity bouts were defined using pre-established criteria, as described in the materials and methods. AU = arbitrary units.

In addition to this reduction in total activity, the diurnal wheel-running rhythms of *Grm2/3* ^-/-^ mice were more fragmented than those of *Grm2/3* ^+/+^ mice. *Grm2/3* ^-/-^ mice engaged in more daily activity bouts (*M* = 4.4) than *Grm2/3* ^+/+^ mice (*M* = 3.5), resulting in a main effect of genotype on the number of daily activity bouts (*F*_1,43_ = 5.071, P = 0.029; Fig 5A). Moreover, the average duration of these bouts was shorter in *Grm2/3* ^-/-^ mice (*M* = 114 min) than *Grm2/3* ^+/+^ mice (*M* = 175 min). Hence, there was also a main effect of genotype on activity bout duration (*F*_1,43_ = 9.137, P = 0.004; Fig 5B).

The diurnal wheel-running rhythms of *Grm2/3* ^-/-^ mice were also less consistent from one day to the next. Interdaily stability was much lower in *Grm2/3* ^-/-^ mice than *Grm2/3* ^+/+^ mice (*F*_1,45_ = 10.094, P = 0.003; Fig 5C), while chi-square periodogram amplitude was also reduced in *Grm2/3* ^-/-^ mice (*F*_1,45_ = 4.722, P = 0.035; Fig 5D). In addition, onset tau error—the degree of variability in daily activity onset—was greater in *Grm2/3* ^-/-^ than *Grm2/3* ^+/+^ mice (*F*_1,45_ = 3.417, P = 0.071).

Period length under 12:12 LD was unaffected by genotype (*F*_1,45_ = 0.010, P = 0.922). Likewise, *Grm2/3* ^+/+^ and *Grm2/3* ^-/-^ mice were equally able to adjust to a shift in the light/dark cycle; following a 6 h phase advance in 12:12 LD, genotype had no effect on the number of days required for re-entrainment (*F*_1,21_ = 0.494, *P* = 0.490).

#### Video-tracking activity analyses

When mice were housed in cages lacking running wheels, and locomotor activity was determined with near-infrared cameras, the rest-activity profiles of *Grm2/3* ^-/-^ mice were very different from those derived using running wheels (see Fig 3D). As with running wheels, light phase activity was greater in *Grm2/3* ^-/-^ mice than *Grm2/3* ^+/+^ mice (*F*_1,22_ = 6.166, P = 0.021; Fig 3E), although the percentage of total daily activity which occurred during the light phase did not vary according to genotype (*F*_1,21_ = 0.625, P = 0.438). Crucially, however, genotype had *no effect* on dark phase activity (*F*_1,21_ = 0.217, P = 0.646; Fig 3F) or total activity levels (*F*_1,21_ = 0.395, P = 0.536). There were no effects or interactions involving recording day for any of these analyses, reflecting the fact that the activity profiles of *Grm2/3* ^+/+^ and *Grm2/3* ^-/-^ mice were relatively consistent across the three recording sessions. Note that specific parameters such as period length cannot be extracted from isolated 24 h video recordings.

#### PIR activity analyses

When mice were housed in cages lacking running wheels, and locomotor activity was determined with PIR motion detectors, the rest-activity profiles of *Grm2/3* ^-/-^ mice were again very different from those derived using running wheels (see Fig 3G). As with running wheels, light phase activity was greater in *Grm2/3* ^-/-^ mice than *Grm2/3* ^+/+^ mice (*F*_1,22_ = 2.551, P = 0.124; Fig 3H), and the percentage of total daily activity which occurred during the light phase was higher in *Grm2/3* ^-/-^ mice (*F*_1,22_ = 3.991, P = 0.058), although neither of these effects reached statistical significance. More importantly, however, genotype had *no effect* on dark phase activity (*F*_1,22_ = 0.606, P = 0.445; Fig 3I) or total activity levels (*F*_1,22_ = 0.122, P = 0.730). (Note that mean values for all PIR analyses are included in Table 2, and representative actograms of 12:12 LD are depicted in Fig 4B).

**Table 2:** Descriptive statistics for selected rest-activity parameters derived from 14 consecutive days of passive-infrared (PIR) data.

Parameter	*Grm2/3* ^+/+^ (mean ± SEM)	*Grm2/3* ^-/-^ (mean ± SEM)	p-value
Daily activity (AU)	2563 ± 209 (*n* = 12)	2470 ± 163 (*n* = 12)	0.730
Dark phase activity (AU)	2213 ± 194 (*n* = 12)	2032 ± 128 (*n* = 12)	0.445
Light phase activity (AU)	350 ± 19 (*n* = 12)	438 ± 52 (*n* = 12)	0.124
Relative light phase activity (%)	14.2 ± 0.9 (*n* = 12)	17.4 ± 1.4 (*n* = 12)	0.058
Daily activity bouts	12.6 ± 0.5 (*n* = 12)	11.1 ± 0.4 (*n* = 12)	0.034
Activity bout duration (min)	66 ± 4 (*n* = 12)	86 ± 5 (*n* = 12)	0.006
Interdaily stability (AU)	0.72 ± 0.02 (*n* = 12)	0.77 ± 0.02 (*n* = 12)	0.085
Chi-square periodogram amplitude (AU)	1653 ± 115 (*n* = 12)	1627 ± 83 (*n* = 12)	0.855
Onset tau error (h)	0.49 ± 0.06 (*n* = 12)	0.60 ± 0.07 (*n* = 12)	0.224
Period length (h)	23.95 ± 0.01 (*n* = 12)	23.97 ± 0.02 (*n* = 12)	0.372

Mice were housed under a 12:12 h light/dark (12:12 LD) cycle at 100 lux. Units of measurement and sample sizes are indicated in brackets. AU = arbitrary units. SEM = standard error of the mean.

Notably, there was no evidence that the diurnal rest-activity rhythms of *Grm2/3* ^-/-^ mice were less consistent than those of *Grm2/3* ^+/+^ mice; chi-square periodogram amplitude (*F*_1,22_ = 0.034, P = 0.855; Fig 5H) and onset tau error (*F*_1,22_ = 1.563, P = 0.224) were both unaffected by genotype. Moreover, interdaily stability—which was significantly *reduced* in *Grm2/3* ^-/-^ mice in the wheel-running dataset—was numerically *increased* in *Grm2/3* ^-/-^ mice in the PIR-derived dataset (*F*_1,22_ = 3.248, P = 0.085; Fig 5G). A similar picture emerged for activity fragmentation; whereas activity fragmentation was significantly *increased* in *Grm2/3* ^-/-^ mice in the wheel-running dataset, it was significantly *reduced* in *Grm2/3* ^-/-^ mice in the PIR-derived dataset. *Grm2/3* ^-/-^ mice engaged in fewer activity bouts than *Grm2/3* ^+/+^ mice (*F*_1,22_ = 5.131, P = 0.034; Fig 5E), while average bout duration was longer in *Grm2/3* ^-/-^ mice (*F*_1,22_ = 9.391, P = 0.006; Fig 5F). As in the wheel-running screen, period length was unaffected by genotype (*F*_1,22_ = 0.832, P = 0.372).

## Discussion

The present study investigated sleep and circadian rhythms in *Grm2/3* double knockout (*Grm2/3* ^-/-^) mice and wildtype (*Grm2/3* ^+/+^) controls. We report three novel findings. Firstly, *Grm2/3* ^-/-^ mice displayed a decrease in immobility-determined sleep time and an increase in immobility-determined sleep fragmentation. Secondly, *Grm2/3* ^-/-^ mice demonstrated heightened sensitivity to the circadian effects of light, as determined using running wheels. More specifically, they showed increased period lengthening in LL, and greater phase delays in response to type I and type II phase-shifting light pulses. Thirdly, the wheel-running activity of *Grm2/3* ^-/-^ mice was significantly perturbed under a standard 12:12 LD cycle.

### Reduced sleep time and increased sleep fragmentation in *Grm2/3* ^-/-^ mice

Our observation of reduced sleep time in *Grm2/3* ^-/-^ mice is consistent with reports of reduced sleep in wildtype rats following the administration of various mGlu2/3-modulating drugs [43–50]. In these pharmacological models, reduced sleep time often reflects the selective suppression of REM sleep. Somewhat counter-intuitively, REM sleep is reduced by mGlu2/3 agonists, antagonists, positive allosteric modulators and negative allosteric modulators alike [43–50]. Crucially, the mGlu2/3 agonist LY354740 inhibits REM sleep in wildtype mice but not in *Grm2* ^-/-^ mice, which implies that mGlu2 contributes to these drug effects [43].

In which brain region(s) do group II metabotropic glutamate receptors exert their influence on REM sleep? The amygdala is a promising candidate, since it is known to influence sleep regulation [69–74], and *Grm2* is highly expressed in this region [26, 28, 30]. Indeed, Dong and colleagues observed reduced REM sleep in wildtype rats following microinjection of the mGlu2/3 agonist LY379268 directly into the basal amygdala [46]. Another region of interest is the thalamic reticular nucleus, which forms part of the ascending arousal system; mGlu2/3 antagonists are known to modulate the inhibitory output of this structure [51, 52].

In addition to reduced sleep time, *Grm2/3* ^-/-^ mice exhibited an increase in sleep fragmentation. Consistent with this observation, both the mGlu2/3 antagonist LY341495 and the mGlu2 negative allosteric modulator RO4491533 increase the number of transitions between sleep and wakefulness in wildtype rats [44]. Hence, group II metabotropic glutamate receptors may be involved in the putative ‘flip-flop’ circuit involving the ventrolateral preoptic nuclei that is hypothesised to control sleep onset and offset [75]. The inability of *Grm2/3* ^-/-^ mice to remain asleep for sustained periods might also explain the overall reduction in their sleep time.

### mGlu2 and/or mGlu3 modulate the sensitivity of the circadian system to light

Period length in LL was significantly greater in *Grm2/3* ^-/-^ mice than *Grm2/3* ^+/+^ mice, despite the fact that period length was unaffected by genotype in DD. Moreover, following both type I and type II phase-shifting light pulses, phase delays were greater in *Grm2/3* ^-/-^ mice than *Grm2/3* ^+/+^ mice. Negative masking during the type II pulse was unaffected by genotype, although this may reflect a ceiling effect, since the suppression of wheel-running activity was very high in mice of both genotypes. Consistent with our observations in *Grm*2/3^-/-^ mice, light-induced phase delays in wildtype C57Bl/6J mice were enhanced by the administration of the mGlu2/3 negative allosteric modulator RO4432717. While it has previously been reported that mGlu2/3-modulating drugs can influence the magnitude of light-induced phase delays in wildtype hamsters [41, 42], our own data represent the first demonstration of such an effect in wildtype mice. It is also significant that we obtained analogous results in a transgenic model and an acute pharmacological model; this suggests that the heightened light sensitivity of the *Grm2/3* ^-/-^ mouse is not a consequence of altered neurodevelopment.

Collectively, these results demonstrate that mGlu2 and/or mGlu3 can modulate the sensitivity of the circadian system to light, which raises the possibility that mGlu2/3-targeting compounds may be useful for the treatment of circadian rhythm disorders. The relevance of these findings to schizophrenia is unclear, however. In humans, the suppression of nocturnal melatonin secretion by light is the standard measure of circadian photosensitivity; there is some evidence that this response is upregulated in bipolar disorder and seasonal affective disorder [76], but such studies have not been undertaken in individuals with schizophrenia.

It is possible that the enhanced light-induced phase delays shown by *Grm2/3* ^-/-^ mice reflect the absence of mGlu2 and/or mGlu3 from RHT-SCN synapses. Photic entrainment cues are transmitted from the eye to the SCN via the axons of photosensitive retinal ganglion cells, which together make up the RHT [77, 78]. While ionotropic glutamate receptors *mediate* the transduction of light information from the retina to the SCN via the RHT [79–84], metabotropic glutamate receptors may play a *modulatory* role at RHT-SCN synapses [40–42]. Consistent with the fact that group II metabotropic glutamate receptors are presynaptic inhibitory autoreceptors, mGlu2/3 agonists serve to inhibit NMDA-evoked calcium influx in SCN neurons [40], presumably via a reduction in presynaptic glutamate release. By the same logic, the antagonism or deletion of group II metabotropic glutamate receptors might be expected to *increase* presynaptic glutamate release at RHT-SCN synapses, explaining the enhanced light-induced phase delays witnessed in *Grm2/3* ^-/-^ mice and in wildtype C57Bl/6J mice following the administration of RO4432717. It should also be noted that *Grm2* is expressed in the retina, in cholinergic amacrine cells [85–87], although its role here is not well understood. *Grm3* is not expressed in the mammalian retina [85–87].

### Origins and implications of the perturbed wheel-running activity of *Grm2/3* ^-/-^ mice

Under 12:12 LD, the diurnal wheel-running rhythms of *Grm2/3* ^-/-^ mice were characterised by increased activity fragmentation and reduced consistency, while total activity levels were substantially reduced. By contrast, general home-cage activity levels—assessed with both near-infrared cameras and PIR motion detectors—were unchanged in *Grm2/3* ^-/-^ mice. The consistency of home-cage diurnal rest-activity rhythms, as measured with PIR motion detectors, was also unaffected by genotype. This implies that the perturbed diurnal wheel-running rhythms of *Grm2/3* ^-/-^ mice are in some way related to the use of the wheels themselves.

There are several possible reasons for the significantly reduced wheel-running activity of *Grm2/3* ^-/-^ mice, which are not mutually exclusive. It could reflect heightened object neophobia—a manifestation of increased anxiety—since all mice were housed in empty home-cages prior to the introduction of running wheels. This seems unlikely, however, given that *Grm2/3* ^+/+^ and *Grm2/3* ^-/-^ mice showed equal exploration of another novel object (a transparent play-tube) immediately after its introduction to the home-cage (see S1 Fig). Moreover, a previous study found no evidence of altered anxiety in *Grm2/3* ^-/-^ mice in either the elevated plus maze, open field, black and white alley, or novelty-suppressed feeding tasks [54]. A more straightforward explanation is a deficit in motor function. In support of this hypothesis, *Grm2/3* ^-/-^ mice demonstrate mild but significant impairments in standard tests of motor coordination; they are impaired at both the accelerating rotarod task and the multiple static rods task [54]. A final possibility is that *Grm2/3* ^-/-^ mice are less *motivated* to engage in wheel-running activity; perhaps the physical act of wheel-running is inherently less rewarding for *Grm2/3* ^-/-^ than *Grm2/3* ^+/+^ mice. Long-term voluntary wheel-running is known to be rewarding for rodents, and produces plastic changes in mesolimbic reward neurocircuity [88]. Significantly, striatal dopamine is reduced in *Grm2/3* ^-/-^ mice, particularly in the nucleus accumbens [89], a brain region involved in reward and motivation [90, 91].

It is somewhat more difficult to explain why the PIR-derived diurnal rest-activity rhythms of *Grm2/3* ^-/-^ mice were *less* fragmented than those of *Grm2/3* ^+/+^ mice, when their diurnal wheel-running rhythms were *more* fragmented. By contrast, *Grm2/3* ^-/-^ mice demonstrated greater light phase activity than *Grm2/3* ^+/+^ mice in all three assays (i.e. wheel-running, PIR *and* video-tracking). This is likely a direct consequence of their reduced light phase sleep time.

Interestingly, our results deviate slightly from those of previous experiments that have studied diurnal rest-activity rhythms in *Grm2/3* ^-/-^ mice. Lyon and colleagues observed that *Grm2/3* ^-/-^ mice were less active than *Grm2/3* ^+/+^ mice in cages lacking running wheels, particularly in the dark phase [55]. These results should be treated with caution, however, as activity was assessed over a relatively short period (less than 3 light/dark cycles), and recording began immediately after transferring the mice from the familiar home-cage environment to a novel activity-monitoring cage [55]. Furthermore, mice in this experiment were group-housed in the home-cage prior to single-housing in the activity-monitoring cages [55]. By contrast, mice were singly-housed throughout the present study.

### Relative merits of wheel-running, video-tracking and PIR assays

The *Grm2/3* ^-/-^ mouse clearly demonstrates that the rest-activity phenotype of a mouse can vary markedly depending on the method used to assay locomotor behaviour. This is perhaps unsurprising given that wheel-running is a measure of voluntary exercise rather than general home-cage activity. Indeed, wheel-running is a complex behaviour that may be subject to multiple influences including arousal, motivation, anxiety, motor coordination, and sensitivity to reward [92–94]. Hence, wheel-running might not be the most appropriate assay for disease-relevant mouse models that show alterations in one or more of these parameters.

The present study is the first to use PIR motion detectors to simultaneously assess sleep and diurnal rest-activity rhythms in a transgenic mouse. The use of PIR motion detectors for sleep estimation was recently validated with simultaneous EEG-based sleep determination in wildtype mice [Brown *et al*., *in review*]. The similarity of the PIR-derived and video-derived sleep data presented here serves as further validation of this novel technique. PIR assays offer two main advantages over video-tracking assays: firstly, they enable the estimation of sleep over a longer period of time, providing a more representative view of an animal’s sleep profile; and secondly, they enable the computation of specific parameters that cannot be extracted from isolated 24 h video recordings, such as period length.

### Sleep disruption in neuropsychiatric disorders

As outlined previously, up to 80% of schizophrenia patients are afflicted by SCRD [2]. The prevalence of SCRD in schizophrenia has been attributed to the use of antipsychotic medication and the absence of social routine [10, 11], but, contrary to these explanations, SCRD is seen in both medication-naïve patients [11] and patients that follow a fixed daily routine [7]. Instead, the co-morbidity of schizophrenia and SCRD may reflect dysfunction in common brain mechanisms, such as the glutamate system, which is clearly relevant to sleep [23–25], photic entrainment [14–16] *and* schizophrenia [12, 13]. At the receptor level, multiple lines of evidence implicate group II metabotropic glutamate receptors in sleep regulation and photic entrainment [14, 39–52], while there is also a robust association between the *Grm3* locus and schizophrenia [32–36]. The observation of reduced and fragmented sleep in *Grm2/3* ^-/-^ mice implies—but does not prove—that abnormal group II metabotropic glutamate receptor signalling may be relevant to sleep disruption in schizophrenia. Having said this, it is important to acknowledge that the *Grm2/3* ^-/-^ mouse is not a ‘disease model’ in itself; although there is evidence for the altered dimerisation of mGlu3 in schizophrenia [53], neither mGlu2 or mGlu3 are completely absent in the brains of schizophrenia patients.

Group II metabotropic glutamate receptors could be relevant to SCRD in neuropsychiatric disorders besides schizophrenia. *Grm3* is also associated with bipolar disorder [95, 96], and, in contrast to schizophrenia, reduced REM sleep is very common in this condition [97]. This is significant given that REM sleep is reduced in wildtype rats after the administration of various mGlu2/3-modulating drugs [43–50]. Moreover, there is evidence that circadian photosensitivity is elevated in bipolar disorder [76], just as it is in *Grm2/3* ^-/-^ mice.

### Conclusions

The results of the present study add to a growing body of evidence implicating group II metabotropic glutamate receptors in both photic entrainment and sleep regulation pathways. Consistent with the effects of a wide range of mGlu2/3-modulating drugs, we provide the first evidence that the genetic ablation of mGlu2 & 3 increases sleep fragmentation and reduces sleep time in mice. Our findings are also the first to demonstrate that the genetic ablation of mGlu2 & 3 heightens the sensitivity of the circadian system to light. These observations are compatible with our hypothesis that SCRD and specific neuropsychiatric disorders share common mechanistic origins [10, 11], and tentatively suggest that altered group II metabotropic glutamate receptor signalling could be relevant to sleep disruption in schizophrenia, and to sleep and circadian disruption in bipolar disorder. In addition, we report that the rest-activity phenotype of *Grm2/3* ^-/-^ mice is critically dependent on the method used to assay locomotor behaviour. We argue that wheel-running might not be the most appropriate assay for disease-relevant mouse models with altered arousal, motivation, anxiety, motor coordination and/or sensitivity to reward, since each of these parameters has the potential to influence wheel-running activity.

## Supporting Information

S1 Fig Home-cage object interaction is unaltered in *Grm2/3* ^-/-^ mice. Genotype has no effect on interaction with a novel object during the first 10 min (A) or first hour (B) after its introduction to the home-cage (at ZT16), or during the entirety of the subsequent dark phase (C). The object was a transparent acrylic play-tube. Object interaction was defined as the amount of time spent in contact with the play-tube (including time spent within it).
(TIF)

S1 Table NC3Rs ARRIVE Guidelines Checklist. (PDF)

## References

[1] van Os J, Kapur S. Schizophrenia. The Lancet. 2009;374(9690):635–45. 10.1016/S0140-6736(09)60995-8 19700006

[2] Cohrs S. Sleep disturbances in patients with schizophrenia: impact and effect of antipsychotics. CNS drugs. 2008;22(11):939–62. 18840034 10.2165/00023210-200822110-00004

[3] Manoach DS, Stickgold R. Does abnormal sleep impair memory consolidation in schizophrenia? Frontiers in human neuroscience. 2009;3:21 10.3389/neuro.09.021.2009 19750201 PMC2741296

[4] Monti JM, BaHammam AS, Pandi-Perumal SR, Bromundt V, Spence DW, Cardinali DP, et al Sleep and circadian rhythm dysregulation in schizophrenia. Prog Neuropsychopharmacol Biol Psychiatry. 2013;43:209–16. 10.1016/j.pnpbp.2012.12.021 23318689

[5] Martin J, Jeste DV, Caliguiri MP, Patterson T, Heaton R, Ancoli-Israel S. Actigraphic estimates of circadian rhythms and sleep/wake in older schizophrenia patients. Schizophr Res. 2001;47(1):77–86. 11163547 10.1016/s0920-9964(00)00029-3 PMC2758687

[6] Martin JL, Jeste DV, Ancoli-Israel S. Older schizophrenia patients have more disrupted sleep and circadian rhythms than age-matched comparison subjects. J Psychiatr Res. 2005;39(3):251–9. 15725423 10.1016/j.jpsychires.2004.08.011

[7] Wulff K, Dijk DJ, Middleton B, Foster RG, Joyce EM. Sleep and circadian rhythm disruption in schizophrenia. Br J Psychiatry. 2012;200(4):308–16. 10.1192/bjp.bp.111.096321 22194182 PMC3317037

[8] Wulff K, Joyce E, Middleton B, Dijk DJ, Foster RG. The suitability of actigraphy, diary data, and urinary melatonin profiles for quantitative assessment of sleep disturbances in schizophrenia: a case report. Chronobiology international. 2006;23(1–2):485–95. 16687321 10.1080/07420520500545987

[9] Wulff K, Porcheret K, Cussans E, Foster RG. Sleep and circadian rhythm disturbances: multiple genes and multiple phenotypes. Current opinion in genetics & development. 2009;19(3):237–46. 19423332 10.1016/j.gde.2009.03.007

[10] Pritchett D, Wulff K, Oliver PL, Bannerman DM, Davies KE, Harrison PJ, et al Evaluating the links between schizophrenia and sleep and circadian rhythm disruption. J Neural Transm. 2012;119(10):1061–75. 22569850 10.1007/s00702-012-0817-8

[11] Wulff K, Gatti S, Wettstein JG, Foster RG. Sleep and circadian rhythm disruption in psychiatric and neurodegenerative disease. Nat Rev Neurosci. 2010;11(8):589–99. 10.1038/nrn2868 20631712

[12] Tamminga CA. Schizophrenia and glutamatergic transmission. Critical reviews in neurobiology. 1998;12(1–2):21–36. 9444480 10.1615/critrevneurobiol.v12.i1-2.20

[13] Tsai G, Coyle JT. Glutamatergic mechanisms in schizophrenia. Annual review of pharmacology and toxicology. 2002;42:165–79. 11807169 10.1146/annurev.pharmtox.42.082701.160735

[14] Ebling FJ. The role of glutamate in the photic regulation of the suprachiasmatic nucleus. Progress in neurobiology. 1996;50(2–3):109–32. 8971980 10.1016/s0301-0082(96)00032-9

[15] Reppert SM, Weaver DR. Molecular analysis of mammalian circadian rhythms. Annual review of physiology. 2001;63:647–76. 11181971 10.1146/annurev.physiol.63.1.647

[16] Reppert SM, Weaver DR. Coordination of circadian timing in mammals. Nature. 2002;418(6901):935–41. 12198538 10.1038/nature00965

[17] Michel S, Marek R, Vanderleest HT, Vansteensel MJ, Schwartz WJ, Colwell CS, et al Mechanism of bilateral communication in the suprachiasmatic nucleus. Eur J Neurosci. 2013;37(6):964–71. 10.1111/ejn.12109 23311402

[18] Cui LN, Coderre E, Renaud LP. Glutamate and GABA mediate suprachiasmatic nucleus inputs to spinal-projecting paraventricular neurons. American journal of physiology Regulatory, integrative and comparative physiology. 2001;281(4):R1283–9. 11557637 10.1152/ajpregu.2001.281.4.R1283

[19] Hermes ML, Coderre EM, Buijs RM, Renaud LP. GABA and glutamate mediate rapid neurotransmission from suprachiasmatic nucleus to hypothalamic paraventricular nucleus in rat. The Journal of physiology. 1996;496(3):749–57. 8930841 10.1113/jphysiol.1996.sp021724 PMC1160861

[20] Saint-Mleux B, Bayer L, Eggermann E, Jones BE, Muhlethaler M, Serafin M. Suprachiasmatic modulation of noradrenaline release in the ventrolateral preoptic nucleus. J Neurosci. 2007;27(24):6412–6. 17567801 10.1523/JNEUROSCI.1432-07.2007 PMC6672428

[21] Sun X, Whitefield S, Rusak B, Semba K. Electrophysiological analysis of suprachiasmatic nucleus projections to the ventrolateral preoptic area in the rat. Eur J Neurosci. 2001;14(8):1257–74. 11703455 10.1046/j.0953-816x.2001.0001755.x

[22] Zhang L, Kolaj M, Renaud LP. Suprachiasmatic nucleus communicates with anterior thalamic paraventricular nucleus neurons via rapid glutamatergic and gabaergic neurotransmission: state-dependent response patterns observed in vitro. Neuroscience. 2006;141(4):2059–66. 16797851 10.1016/j.neuroscience.2006.05.042

[23] Kodama T, Lai YY, Siegel JM. Enhanced glutamate release during REM sleep in the rostromedial medulla as measured by in vivo microdialysis. Brain Res. 1998;780(1):178–81. 9497097 PMC8848830

[24] Lopez-Rodriguez F, Medina-Ceja L, Wilson CL, Jhung D, Morales-Villagran A. Changes in extracellular glutamate levels in rat orbitofrontal cortex during sleep and wakefulness. Archives of medical research. 2007;38(1):52–5. 17174723 10.1016/j.arcmed.2006.07.004

[25] Naylor E, Aillon DV, Gabbert S, Harmon H, Johnson DA, Wilson GS, et al Simultaneous real-time measurement of EEG/EMG and l-glutamate in mice: A biosensor study of neuronal activity during sleep. Journal of Electroanalytical Chemistry. 2011;656(1–2):106–13. 27076812 10.1016/j.jelechem.2010.12.031 PMC4827928

[26] Gu G, Lorrain DS, Wei H, Cole RL, Zhang X, Daggett LP, et al Distribution of metabotropic glutamate 2 and 3 receptors in the rat forebrain: Implication in emotional responses and central disinhibition. Brain Res. 2008;1197:47–62. 10.1016/j.brainres.2007.12.057 18242587

[27] Makoff A, Volpe F, Lelchuk R, Harrington K, Emson P. Molecular characterization and localization of human metabotropic glutamate receptor type 3. Brain research Molecular brain research. 1996;40(1):55–63. 8840013 10.1016/0169-328x(96)00037-x

[28] Ohishi H, Shigemoto R, Nakanishi S, Mizuno N. Distribution of the mRNA for a metabotropic glutamate receptor (mGluR3) in the rat brain: an in situ hybridization study. J Comp Neurol. 1993;335(2):252–66. 8227517 10.1002/cne.903350209

[29] Ohnuma T, Augood SJ, Arai H, McKenna PJ, Emson PC. Expression of the human excitatory amino acid transporter 2 and metabotropic glutamate receptors 3 and 5 in the prefrontal cortex from normal individuals and patients with schizophrenia. Brain research Molecular brain research. 1998;56(1–2):207–17. 9602129 10.1016/s0169-328x(98)00063-1

[30] Tamaru Y, Nomura S, Mizuno N, Shigemoto R. Distribution of metabotropic glutamate receptor mGluR3 in the mouse CNS: differential location relative to pre- and postsynaptic sites. Neuroscience. 2001;106(3):481–503. 11591452 10.1016/s0306-4522(01)00305-0

[31] Wright RA, Johnson BG, Zhang C, Salhoff C, Kingston AE, Calligaro DO, et al CNS distribution of metabotropic glutamate 2 and 3 receptors: transgenic mice and [(3)H]LY459477 autoradiography. Neuropharmacology. 2013;66:89–98. 10.1016/j.neuropharm.2012.01.019 22313530

[32] Schizophrenia Working Group of the Psychiatric Genomics C. Biological insights from 108 schizophrenia-associated genetic loci. Nature. 2014;511(7510):421–7. 10.1038/nature13595 25056061 PMC4112379

[33] Egan MF, Straub RE, Goldberg TE, Yakub I, Callicott JH, Hariri AR, et al Variation in GRM3 affects cognition, prefrontal glutamate, and risk for schizophrenia. Proc Natl Acad Sci U S A. 2004;101(34):12604–9. 15310849 10.1073/pnas.0405077101 PMC515104

[34] Harrison PJ, Lyon L, Sartorius LJ, Burnet PW, Lane TA. The group II metabotropic glutamate receptor 3 (mGluR3, mGlu3, GRM3): expression, function and involvement in schizophrenia. J Psychopharmacol. 2008;22(3):308–22. 10.1177/0269881108089818 18541626

[35] Kandaswamy R, McQuillin A, Sharp SI, Fiorentino A, Anjorin A, Blizard RA, et al Genetic association, mutation screening, and functional analysis of a Kozak sequence variant in the metabotropic glutamate receptor 3 gene in bipolar disorder. JAMA Psychiatry. 2013;70(6):591–8. 10.1001/jamapsychiatry.2013.38 23575746

[36] Sartorius LJ, Weinberger DR, Hyde TM, Harrison PJ, Kleinman JE, Lipska BK. Expression of a GRM3 splice variant is increased in the dorsolateral prefrontal cortex of individuals carrying a schizophrenia risk SNP. Neuropsychopharmacology. 2008;33(11):2626–34. 10.1038/sj.npp.1301669 18256595

[37] de Quervain DJ, Papassotiropoulos A. Identification of a genetic cluster influencing memory performance and hippocampal activity in humans. Proc Natl Acad Sci U S A. 2006;103(11):4270–4. 16537520 10.1073/pnas.0510212103 PMC1390747

[38] Vinson PN, Conn PJ. Metabotropic glutamate receptors as therapeutic targets for schizophrenia. Neuropharmacology. 2012;62(3):1461–72. 10.1016/j.neuropharm.2011.05.005 21620876 PMC3189289

[39] Ghosh PK, Baskaran N, van den Pol AN. Developmentally regulated gene expression of all eight metabotropic glutamate receptors in hypothalamic suprachiasmatic and arcuate nuclei—a PCR analysis. Brain research Developmental brain research. 1997;102(1):1–12. 9298229 10.1016/s0165-3806(97)00066-7

[40] Haak LL. Metabotropic glutamate receptor modulation of glutamate responses in the suprachiasmatic nucleus. Journal of neurophysiology. 1999;81(3):1308–17. 10085357 10.1152/jn.1999.81.3.1308

[41] Gannon RL, Millan MJ. Positive and negative modulation of circadian activity rhythms by mGluR5 and mGluR2/3 metabotropic glutamate receptors. Neuropharmacology. 2011;60(2–3):209–15. 20831878 10.1016/j.neuropharm.2010.08.022

[42] Haak LL, Albers HE, Mintz EM. Modulation of photic response by the metabotropic glutamate receptor agonist t-ACPD. Brain Res Bull. 2006;71(1–3):97–100. 17113934 10.1016/j.brainresbull.2006.08.006 PMC1771113

[43] Ahnaou A, Dautzenberg FM, Geys H, Imogai H, Gibelin A, Moechars D, et al Modulation of group II metabotropic glutamate receptor (mGlu2) elicits common changes in rat and mice sleep-wake architecture. Eur J Pharmacol. 2009;603(1–3):62–72. 10.1016/j.ejphar.2008.12.020 19046965

[44] Ahnaou A, Ver Donck L, Drinkenburg WH. Blockade of the metabotropic glutamate (mGluR2) modulates arousal through vigilance states transitions: evidence from sleep-wake EEG in rodents. Behav Brain Res. 2014;270:56–67. 10.1016/j.bbr.2014.05.003 24821401

[45] Cid JM, Tresadern G, Vega JA, de Lucas AI, Matesanz E, Iturrino L, et al Discovery of 3-cyclopropylmethyl-7-(4-phenylpiperidin-1-yl)-8-trifluoromethyl[1,2,4]triazolo[4, 3-a]pyridine (JNJ-42153605): a positive allosteric modulator of the metabotropic glutamate 2 receptor. J Med Chem. 2012;55(20):8770–89. 10.1021/jm3010724 23072213

[46] Dong E, Wellman LL, Yang L, Sanford LD. Effects of microinjections of Group II metabotropic glutamate agents into the amygdala on sleep. Brain Res. 2012;1452:85–95. 10.1016/j.brainres.2012.03.003 22453124 PMC3326230

[47] Feinberg I, Campbell IG, Schoepp DD, Anderson K. The selective group mGlu2/3 receptor agonist LY379268 suppresses REM sleep and fast EEG in the rat. Pharmacol Biochem Behav. 2002;73(2):467–74. 12117602 10.1016/s0091-3057(02)00843-2

[48] Feinberg I, Schoepp DD, Hsieh KC, Darchia N, Campbell IG. The metabotropic glutamate (mGLU)2/3 receptor antagonist LY341495 [2S-2-amino-2-(1S,2S-2-carboxycyclopropyl-1-yl)-3-(xanth-9-yl)propanoic acid] stimulates waking and fast electroencephalogram power and blocks the effects of the mGLU2/3 receptor agonist ly379268 [(-)-2-oxa-4-aminobicyclo[3.1.0]hexane-4,6-dicarboxylate] in rats. J Pharmacol Exp Ther. 2005;312(2):826–33. 15383637 10.1124/jpet.104.076547

[49] Fell MJ, Witkin JM, Falcone JF, Katner JS, Perry KW, Hart J, et al N-(4-((2-(trifluoromethyl)-3-hydroxy-4-(isobutyryl)phenoxy)methyl)benzyl)-1-methy l-1H-imidazole-4-carboxamide (THIIC), a novel metabotropic glutamate 2 potentiator with potential anxiolytic/antidepressant properties: in vivo profiling suggests a link between behavioral and central nervous system neurochemical changes. J Pharmacol Exp Ther. 2011;336(1):165–77. 10.1124/jpet.110.172957 20947638

[50] Lavreysen H, Langlois X, Ahnaou A, Drinkenburg W, te Riele P, Biesmans I, et al Pharmacological characterization of JNJ-40068782, a new potent, selective, and systemically active positive allosteric modulator of the mGlu2 receptor and its radioligand [3H]JNJ-40068782. J Pharmacol Exp Ther. 2013;346(3):514–27. 10.1124/jpet.113.204990 23766542

[51] Salt TE, Turner JP. Modulation of sensory inhibition in the ventrobasal thalamus via activation of group II metabotropic glutamate receptors by 2R,4R-aminopyrrolidine-2,4-dicarboxylate. Experimental brain research. 1998;121(2):181–5. 9696387 10.1007/s002210050450

[52] Turner JP, Salt TE. Group II and III metabotropic glutamate receptors and the control of the nucleus reticularis thalami input to rat thalamocortical neurones in vitro. Neuroscience. 2003;122(2):459–69. 14614910 10.1016/j.neuroscience.2003.08.014

[53] Corti C, Crepaldi L, Mion S, Roth AL, Xuereb JH, Ferraguti F. Altered dimerization of metabotropic glutamate receptor 3 in schizophrenia. Biol Psychiatry. 2007;62(7):747–55. 17531207 10.1016/j.biopsych.2006.12.005

[54] De Filippis B, Lyon L, Taylor A, Lane T, Burnet PW, Harrison PJ, et al The role of group II metabotropic glutamate receptors in cognition and anxiety: Comparative studies in *GRM2-/-*, *GRM3-/-* and *GRM2/3-/-* knockout mice. Neuropharmacology. 2015;89:19–32. 10.1016/j.neuropharm.2014.08.010 25158312 PMC4259517

[55] Lyon L, Burnet PW, Kew JN, Corti C, Rawlins JN, Lane T, et al Fractionation of spatial memory in GRM2/3 (mGlu2/mGlu3) double knockout mice reveals a role for group II metabotropic glutamate receptors at the interface between arousal and cognition. Neuropsychopharmacology. 2011;36(13):2616–28. 10.1038/npp.2011.145 21832989 PMC3230485

[56] Lyon L, Kew JN, Corti C, Harrison PJ, Burnet PW. Altered hippocampal expression of glutamate receptors and transporters in GRM2 and GRM3 knockout mice. Synapse. 2008;62(11):842–50. 10.1002/syn.20553 18720515 PMC2673354

[57] Yokoi M, Kobayashi K, Manabe T, Takahashi T, Sakaguchi I, Katsuura G, et al Impairment of hippocampal mossy fiber LTD in mice lacking mGluR2. Science. 1996;273(5275):645–7. 8662555 10.1126/science.273.5275.645

[58] Corti C, Battaglia G, Molinaro G, Riozzi B, Pittaluga A, Corsi M, et al The use of knock-out mice unravels distinct roles for mGlu2 and mGlu3 metabotropic glutamate receptors in mechanisms of neurodegeneration/neuroprotection. J Neurosci. 2007;27(31):8297–308. 17670976 10.1523/JNEUROSCI.1889-07.2007 PMC6673047

[59] Pilorz V, Steinlechner S, Oster H. Age and oestrus cycle-related changes in glucocorticoid excretion and wheel-running activity in female mice carrying mutations in the circadian clock genes Per1 and Per2. Physiology & behavior. 2009;96(1):57–63. 18786554 10.1016/j.physbeh.2008.08.010

[60] Fisher SP, Godinho SI, Pothecary CA, Hankins MW, Foster RG, Peirson SN. Rapid assessment of sleep-wake behavior in mice. J Biol Rhythms. 2012;27(1):48–58. 10.1177/0748730411431550 22306973 PMC4650254

[61] Mrosovsky N. Masking: history, definitions, and measurement. Chronobiology international. 1999;16(4):415–29. 10442236 10.3109/07420529908998717

[62] Van Someren EJ, Swaab DF, Colenda CC, Cohen W, McCall WV, Rosenquist PB. Bright light therapy: improved sensitivity to its effects on rest-activity rhythms in Alzheimer patients by application of nonparametric methods. Chronobiology international. 1999;16(4):505–18. 10442243 10.3109/07420529908998724

[63] Albrecht U, Foster RG. Placing ocular mutants into a functional context: a chronobiological approach. Methods (San Diego, Calif). 2002;28(4):465–77. 12507465 10.1016/s1046-2023(02)00266-9

[64] Jud C, Schmutz I, Hampp G, Oster H, Albrecht U. A guideline for analyzing circadian wheel-running behavior in rodents under different lighting conditions. Biological procedures online. 2005;7:101–16. 16136228 10.1251/bpo109 PMC1190381

[65] Oliver PL, Sobczyk MV, Maywood ES, Edwards B, Lee S, Livieratos A, et al Disrupted circadian rhythms in a mouse model of schizophrenia. Current biology: CB. 2012;22(4):314–9. 10.1016/j.cub.2011.12.051 22264613 PMC3356578

[66] Aschoff J. Circadian rhythms in man. Science. 1965;148(3676):1427–32. 14294139 10.1126/science.148.3676.1427

[67] Albrecht U, Zheng B, Larkin D, Sun ZS, Lee CC. MPer1 and mper2 are essential for normal resetting of the circadian clock. J Biol Rhythms. 2001;16(2):100–4. 11302552 10.1177/074873001129001791

[68] Woltering TJ, Wichmann J, Goetschi E, Knoflach F, Ballard TM, Huwyler J, et al Synthesis and characterization of 1,3-dihydro-benzo[b][1,4]diazepin-2-one derivatives: Part 4. In vivo active potent and selective non-competitive metabotropic glutamate receptor 2/3 antagonists. Bioorg Med Chem Lett. 2010;20(23):6969–74. 10.1016/j.bmcl.2010.09.125 20971004

[69] Jha SK, Ross RJ, Morrison AR. Sleep-related neurons in the central nucleus of the amygdala of rats and their modulation by the dorsal raphe nucleus. Physiology & behavior. 2005;86(4):415–26. 16137725 10.1016/j.physbeh.2005.06.033

[70] Morrison AR, Sanford LD, Ross RJ. The amygdala: a critical modulator of sensory influence on sleep. Biological signals and receptors. 2000;9(6):283–96. 11025335 10.1159/000014652

[71] Sanford LD, Nassar P, Ross RJ, Schulkin J, Morrison AR. Prolactin microinjections into the amygdalar central nucleus lead to decreased NREM sleep. Sleep research online: SRO. 1998;1(3):109–13. 11382866

[72] Sanford LD, Parris B, Tang X. GABAergic regulation of the central nucleus of the amygdala: implications for sleep control. Brain Res. 2002;956(2):276–84. 12445696 10.1016/s0006-8993(02)03552-7

[73] Sanford LD, Yang L, Tang X, Dong E, Ross RJ, Morrison AR. Cholinergic regulation of the central nucleus of the amygdala in rats: effects of local microinjections of cholinomimetics and cholinergic antagonists on arousal and sleep. Neuroscience. 2006;141(4):2167–76. 16843604 10.1016/j.neuroscience.2006.05.064

[74] Tang X, Yang L, Liu X, Sanford LD. Influence of tetrodotoxin inactivation of the central nucleus of the amygdala on sleep and arousal. Sleep. 2005;28(8):923–30. 16218075 10.1093/sleep/28.8.923

[75] Schwartz JR, Roth T. Neurophysiology of sleep and wakefulness: basic science and clinical implications. Current neuropharmacology. 2008;6(4):367–78. 10.2174/157015908787386050 19587857 PMC2701283

[76] Nathan PJ, Burrows GD, Norman TR. Melatonin sensitivity to dim white light in affective disorders. Neuropsychopharmacology. 1999;21(3):408–13. 10457538 10.1016/S0893-133X(99)00018-4

[77] Hattar S, Kumar M, Park A, Tong P, Tung J, Yau KW, et al Central projections of melanopsin-expressing retinal ganglion cells in the mouse. J Comp Neurol. 2006;497(3):326–49. 16736474 10.1002/cne.20970 PMC2885916

[78] Provencio I, Cooper HM, Foster RG. Retinal projections in mice with inherited retinal degeneration: implications for circadian photoentrainment. J Comp Neurol. 1998;395(4):417–39. 9619497 10.1002/(sici)1096-9861(19980615)395:4<417::aid-cne1>3.0.co;2-4

[79] Abe H, Rusak B, Robertson HA. Photic induction of Fos protein in the suprachiasmatic nucleus is inhibited by the NMDA receptor antagonist MK-801. Neuroscience Letters. 1991;127(1):9–12. 1831888 10.1016/0304-3940(91)90881-s

[80] Colwell CS, Foster RG, Menaker M. NMDA receptor antagonists block the effects of light on circadian behavior in the mouse. Brain Res. 1991;554(1–2):105–10. 1834303 10.1016/0006-8993(91)90177-w

[81] Kornhauser JM, Nelson DE, Mayo KE, Takahashi JS. Photic and circadian regulation of c-fos gene expression in the hamster suprachiasmatic nucleus. Neuron. 1990;5(2):127–34. 2116813 10.1016/0896-6273(90)90303-w

[82] Liou SY, Shibata S, Iwasaki K, Ueki S. Optic nerve stimulation-induced increase of release of 3H-glutamate and 3H-aspartate but not 3H-GABA from the suprachiasmatic nucleus in slices of rat hypothalamus. Brain Res Bull. 1986;16(4):527–31. 2872946 10.1016/0361-9230(86)90182-6

[83] Mikkelsen JD, Larsen PJ, Mick G, Vrang N, Ebling FJ, Maywood ES, et al Gating of retinal inputs through the suprachiasmatic nucleus: role of excitatory neurotransmission. Neurochemistry international. 1995;27(3):263–72. 8520465 10.1016/0197-0186(95)00039-b

[84] Tominaga K, Geusz ME, Michel S, Inouye ST. Calcium imaging in organotypic cultures of the rat suprachiasmatic nucleus. Neuroreport. 1994;5(15):1901–5. 7841372 10.1097/00001756-199410000-00015

[85] Brandstätter JH, Koulen P, Wässle H. Diversity of glutamate receptors in the mammalian retina. Vision Research. 1998;38(10):1385–97. 9667006 10.1016/s0042-6989(97)00176-4

[86] Hartveit E, Brandstatter JH, Enz R, Wassle H. Expression of the mRNA of seven metabotropic glutamate receptors (mGluR1 to 7) in the rat retina. An in situ hybridization study on tissue sections and isolated cells. Eur J Neurosci. 1995;7(7):1472–83. 7551173 10.1111/j.1460-9568.1995.tb01142.x

[87] Koulen P, Malitschek B, Kuhn R, Wassle H, Brandstatter JH. Group II and group III metabotropic glutamate receptors in the rat retina: distributions and developmental expression patterns. Eur J Neurosci. 1996;8(10):2177–87. 8921309 10.1111/j.1460-9568.1996.tb00739.x

[88] Greenwood BN, Foley TE, Le TV, Strong PV, Loughridge AB, Day HE, et al Long-term voluntary wheel running is rewarding and produces plasticity in the mesolimbic reward pathway. Behav Brain Res. 2011;217(2):354–62. 10.1016/j.bbr.2010.11.005 21070820 PMC3021978

[89] Lane TA, Boerner T, Bannerman DM, Kew JN, Tunbridge EM, Sharp T, et al Decreased striatal dopamine in group II metabotropic glutamate receptor (mGlu2/mGlu3) double knockout mice. BMC neuroscience. 2013;14:102 10.1186/1471-2202-14-102 24053122 PMC3857325

[90] Ikemoto S. Dopamine reward circuitry: two projection systems from the ventral midbrain to the nucleus accumbens-olfactory tubercle complex. Brain research reviews. 2007;56(1):27–78. 17574681 10.1016/j.brainresrev.2007.05.004 PMC2134972

[91] Lammel S, Lim BK, Ran C, Huang KW, Betley MJ, Tye KM, et al Input-specific control of reward and aversion in the ventral tegmental area. Nature. 2012;491(7423):212–7. 10.1038/nature11527 23064228 PMC3493743

[92] Meijer JH, Robbers Y. Wheel running in the wild. Proceedings Biological sciences / The Royal Society. 2014;281(1786). 10.1098/rspb.2014.0210 PMC4046404 24850923

[93] Novak CM, Burghardt PR, Levine JA. The use of a running wheel to measure activity in rodents: relationship to energy balance, general activity, and reward. Neurosci Biobehav Rev. 2012;36(3):1001–14. 10.1016/j.neubiorev.2011.12.012 22230703 PMC4455940

[94] Sherwin CM. Voluntary wheel running: a review and novel interpretation. Animal behaviour. 1998;56(1):11–27. 9710457 10.1006/anbe.1998.0836

[95] Fallin M D, Lasseter Virginia K, Avramopoulos D, Nicodemus Kristin K, Wolyniec Paula S, McGrath John A, et al Bipolar I Disorder and Schizophrenia: A 440–Single-Nucleotide Polymorphism Screen of 64 Candidate Genes among Ashkenazi Jewish Case-Parent Trios. American journal of human genetics. 2005;77(6):918–36. 16380905 10.1086/497703 PMC1285177

[96] Green E, Grozeva D, Norton N, Jones I, Jones L, O’Donovan MC, et al Variation at GRM3 influences susceptibility to psychotic bipolar disorder. American journal of human genetics. 2006;141:697.

[97] Harvey AG, Talbot LS, Gershon A. Sleep Disturbance in Bipolar Disorder Across the Lifespan. Clinical psychology: a publication of the Division of Clinical Psychology of the American Psychological Association. 2009;16(2):256–77. 22493520 10.1111/j.1468-2850.2009.01164.x PMC3321357

