# Supplementary figures and images for: Deletion of Metabotropic Glutamate Receptors 2 and 3 (mGlu2 & mGlu3) in Mice Disrupts Sleep and Wheel-Running Activity, and Increases the Sensitivity of the Circadian System to Light
Source: PLoS One. 2015 May 7;10(5):e0125523. doi: 10.1371/journal.pone.0125523 (PMC4423919; doi:10.1371/journal.pone.0125523)

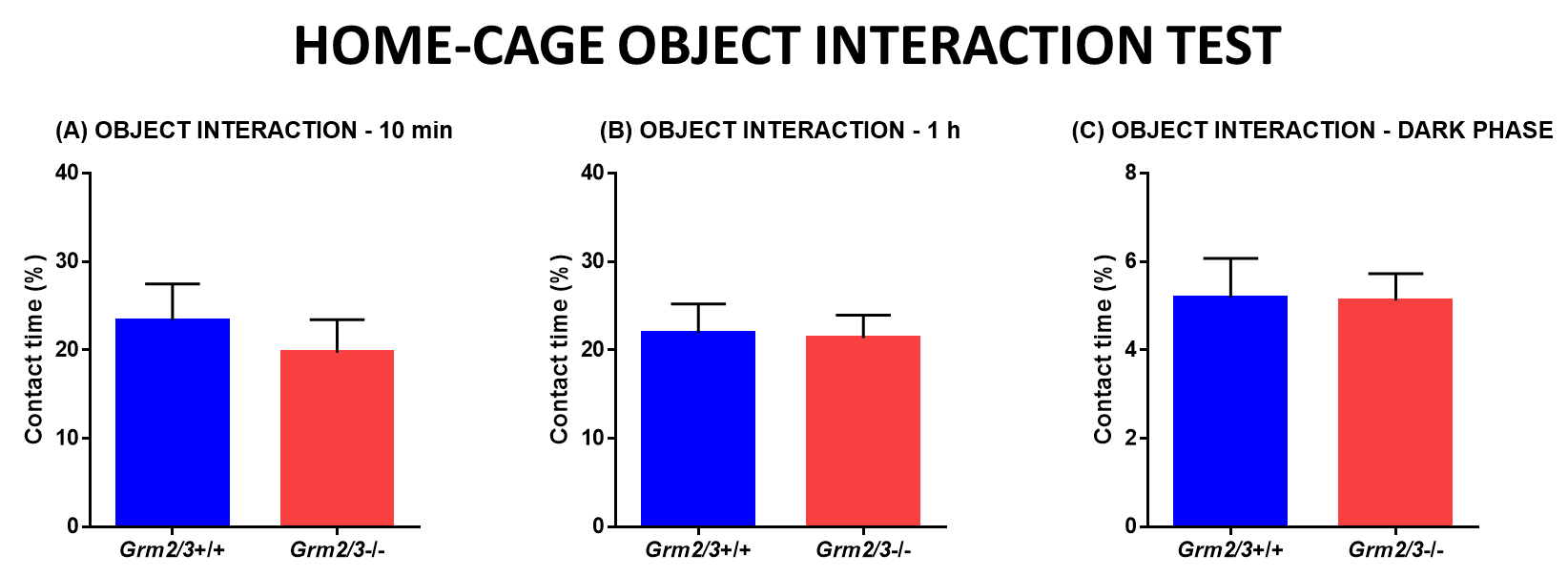

Supplement: S1 Fig — Genotype has no effect on interaction with a novel object during the first 10 min (A) or first hour (B) after its introduction to the home-cage (at ZT16), or during the entirety of the subsequent dark phase (C). The object was a transparent acrylic play-tube. Object interaction was defined as the amount of time spent in contact with the play-tube (including time spent within it). (TIF) [file pone.0125523.s001.tif]
